# Dissociable Role of Corticotropin Releasing Hormone Receptor Subtype 1 on Dopaminergic and D1 Dopaminoceptive Neurons in Cocaine Seeking Behavior

**DOI:** 10.3389/fnbeh.2017.00221

**Published:** 2017-11-13

**Authors:** Rick E. Bernardi, Laura Broccoli, Natalie Hirth, Nicholas J. Justice, Jan M. Deussing, Anita C. Hansson, Rainer Spanagel

**Affiliations:** ^1^Institute of Psychopharmacology, Central Institute of Mental Health, Medical Faculty Mannheim, University of Heidelberg, Mannheim, Germany; ^2^Institute of Molecular Medicine, University of Texas, Houston, TX, United States; ^3^Molecular Neurogenetics, Department of Stress Neurobiology and Neurogenetics, Max Planck Institute of Psychiatry, Munich, Germany

**Keywords:** corticotropin-releasing hormone, corticotropin-releasing factor, inducible knockouts, mesolimbic dopamine system, cocaine sensitization, self-administration, extinction, reinstatement

## Abstract

The ability of many drugs of abuse, including cocaine, to mediate reinforcement and drug-seeking behaviors is in part mediated by the corticotropin-releasing hormone (CRH) system, in which CRH exerts its effects partly via the CRH receptor subtype 1 (CRHR1) in extra-hypothalamic areas. In fact, CRHR1 expressed in regions of the mesolimbic dopamine (DA) system have been demonstrated to modify cocaine-induced DA release and alter cocaine-mediated behaviors. Here we examined the role of neuronal selectivity of CRHR1 within the mesolimbic system on cocaine-induced behaviors. First we used a transgenic mouse line expressing GFP under the control of the Crhr1 promoter for double fluorescence immunohistochemistry to demonstrate the cellular location of CRHR1 in both dopaminergic and D1 dopaminoceptive neurons. We then studied cocaine sensitization, self-administration, and reinstatement in inducible CRHR1 knockouts using the CreERT2/loxP in either dopamine transporter (DAT)-containing neurons (DAT-Crhr1) or dopamine receptor 1 (D1)-containing neurons (D1-Crhr1). For sensitization testing, mice received five daily injections of cocaine (15 mg/kg IP). For self-administration, mice received eight daily 2 h cocaine (0.5 mg/kg per infusion) self-administration sessions followed by extinction and reinstatement testing. There were no differences in the acute or sensitized locomotor response to cocaine in DAT-Crhr1 or D1-Crhr1 mice and their respective controls. Furthermore, both DAT-Crhr1 and D1-Crhr1 mice reliably self-administered cocaine at the level of controls. However, DAT-Crhr1 mice demonstrated a significant increase in cue-induced reinstatement relative to controls, whereas D1-Crhr1 mice demonstrated a significant decrease in cue-induced reinstatement relative to controls. These data demonstrate the involvement of CRHR1 in cue-induced reinstatement following cocaine self-administration, and implicate a bi-directional role of CRHR1 for cocaine craving.

## Introduction

Drug craving and subsequent relapse following abstinence remain major impediments to the treatment of drug addiction in humans. Relapse can be precipitated by one of several factors, including exposure to drug-related cues or the drug itself, as well as stress (Shaham et al., 2003; Sanchis-Segura and Spanagel, 2006). The corticotropin-releasing hormone/corticotropin-releasing factor (CRH/CRF) family of ligands and receptors are important mediators of the neuroendocrine and behavioral response to stress, and hyperfunction of CRH and its type 1 high-affinity receptor (CRHR1) have been implicated in the pathology of chronic stress, including the development of anxiety and mood disorders (Muller and Wurst, 2004; Aubry, 2013). Furthermore, there is high comorbidity of anxiety and mood disorders with substance use disorders in humans (Grant et al., 2004; Bruijnzeel and Gold, 2005), and stress plays an important role in increasing the vulnerability and motivation to abuse drugs in addicted individuals (Koob and Le Moal, 2001, 2008b). Thus, a great deal of preclinical research has been devoted to the link between CRH and CRHR1 in extrahypothalamic brain regions and the effects of drugs of abuse.

CRHR1 is expressed throughout the brain, including the mesolimbic dopamine (DA) system (Van Pett et al., 2000), a brain system extensively demonstrated to mediate drug reinforcement and drug-induced neuroadaptations (Koob and Nestler, 1997; Self, 2004). CRHR1 has been shown to mediate the CRH-induced firing rate of ventral tegmental area (VTA) DA neurons (Wanat et al., 2008), and CRHR1 antagonism has been shown to inhibit striatal DA release (Bagosi et al., 2006) In addition, the activation of CRHR1 by CRH results in an increase in DA release in the nucleus accumbens (Acb; Lemos et al., 2012), and midbrain CRHR1 blockade has been demonstrated to reduce cocaine-induced DA release into the Acb (Lodge and Grace, 2005). CRHR1 is also expressed in DAergic terminal regions including the prefrontal cortex (PFC) and amygdala (Amy; Smagin and Dunn, 2000; Van Pett et al., 2000; Jaferi and Bhatnagar, 2007; Miguel et al., 2014), which mediate excitatory drive and associative processes related to drug-mediated behaviors (Everitt et al., 1999; Peters et al., 2009), as well as regions of the extended amygdala (Van Pett et al., 2000; Kash et al., 2008; Silberman and Winder, 2013), the dysregulation of which also contributes to drug dependence (Koob and Le Moal, 2008b).

Because of the important role of CRHR1 in stress adaptation, the predominance of studies of CRHR1 in drug paradigms have examined its effects on stress-mediated drug-responding. For example, in terms of psychostimulants such as cocaine, peripheral administration of CRHR1 antagonists in rats have been demonstrated to impair a stress-induced increase in the magnitude of cocaine conditioned place preference (Kreibich et al., 2009) and stress-induced reinstatement of cocaine place preference following extinction (Lu et al., 2001; McReynolds et al., 2014). Furthermore, CRH administration induces reinstatement of cocaine-seeking in rats following extinction in the self-administration paradigm (Erb et al., 2006; Brown et al., 2009; Kupferschmidt et al., 2012), and CRHR1 antagonists have been shown to attenuate stress-induced reinstatement of cocaine-seeking in rats (Shaham et al., 1998; but see Wang et al., 2007). Thus, CRHR1 has been demonstrated to play a consistent role in stress-mediated cocaine behaviors.

In addition to its role in stress mediation, the CRH system may contribute to drug reinforcement processes. Behaviorally, CRHR1 antagonists administered peripherally have been demonstrated to reduce cocaine intake in rats during both limited access (Goeders and Guerin, 2000) and escalation (Specio et al., 2008) self-administration procedures. In addition, peripheral CRHR1 antagonism in rats decreased cue responding following cocaine self-administration (Gurkovskaya and Goeders, 2001) and during reinstatement testing following extinction of cocaine self-administration (Goeders and Clampitt, 2002). CRH administered directly into the VTA has been demonstrated to augment cocaine-seeking following self-administration, an effect blocked by a CRHR1 antagonist (Blacktop et al., 2011). Furthermore, Chen et al. (2014) examined the effects of lentiviral knockdown of CRHR1 in the VTA on primary and secondary cocaine reinforcement using self-administration, demonstrating no difference in the acquisition of cocaine self-administration, but a reduced cue-induced cocaine-seeking following an extended abstinence period. Thus, in conjunction with stress mediation, CRHR1 in the reward system likely mediates in general emotional states involved in the motivation for cocaine-taking and cocaine-seeking behaviors.

The studies summarized here demonstrate that CRHR1 plays an important role in the mesolimbic DA system and the response to cocaine. Because CRHR1 are located both presynaptically and postsynaptically in the VTA (Hahn et al., 2009), as well as distributed throughout DAergic terminal regions, its functional role based on site and cellular localization has yet to be characterized. The purpose of the current study was to examine the role of neuronal selectivity of CRHR1 receptors on cocaine-mediated behaviors. To this end, we first examined the cellular location of CRHR1 in both DAergic and D1 dopaminoceptive neurons. Because of the lack of specific immunochemical tools and the low resolution of ligand binding approaches, the anatomical distribution of CRHR1 is not well resolved. An alternative approach is the use of transgenic mice in which the non-mammalian green fluorescent protein (GFP) gene is inserted upstream of the coding region of a target gene and thus is driven by the promoter of this gene. This approach has been successfully applied for anatomical characterization of CRHR1 (Justice et al., 2008). Here, the GFP reporter gene is under the control of the Crhr1 promoter and GFP immunoreactivity (ir) mimics the location of endogenous CRHR1. In this study, we used the Crhr1-GFP transgenic mice to map cellular localization of the receptor within the mesolimbic system. We then generated inducible knockouts of the CRHR1 receptor using the CreERT2/loxP system in either DA transporter (DAT)-containing neurons (DAT-Crhr1) or DA receptor 1 (D1)-containing neurons (D1-Crhr). We first measured locomotion and the acute and sensitized locomotor response to cocaine to determine basal genotypic differences among these inducible knockout lines. We then measured cocaine self-administration and cue-induced reinstatement following extinction. Following cue-induced reinstatement, we also examined yohimbine- and yohimbine + cue-induced reinstatement in the two lines. We show here that despite demonstrating no differences relative to controls in cocaine-induced locomotion or sensitization, and no differences in cocaine SA, resulting from CRHR1 inactivation in DAT- and D1-containing neurons, we found dissociable effects on cue-induced reinstatement. DAT-Crhr1 mice demonstrated a significant increase in cue-induced reinstatement relative to controls, while D1-Crhr1 mice demonstrated a significant decrease in cue-induced reinstatement relative to controls, indicating neuronal selectivity of CRHR1 receptors on dopaminoceptive neurons in the reinstatement of cocaine-seeking.

## Materials and Methods

### Subjects

For immunocytochemistry, three male Crhr1-GFP mice (8–10 weeks) were used. For behavioral experiments, 103 adult male inducible DAT-Crhr1 and D1-Crhr1 mice and their wild-type floxed littermates not carrying the Cre-recombinase (8–10 weeks at the beginning of the experiments) resulting from at least 10 consecutive backcrosses with C57BL/6N were used.

All mice were single-housed in a temperature-controlled (21°C) environment maintained on a 12-h light-dark cycle (lights on at 6 a.m.). Food and water was available *ad libitum*. All experiments were performed in accordance with EU guidelines on the care and use of laboratory animals and were approved by the local animal care committee (Regierungspräsidium, Karlsruhe, Germany). All behavioral testing was conducted during the light phase between 08:00 h and 17:00 h.

### Generation of Mice

We generated transgenic mice expressing GFP under the control of the Crhr1 promoter as previously described (Justice et al., 2008).

The generation of floxed Crhr1 mice has also been previously described (Kühne et al., 2012). Conditional Crhr1 mutant mice were obtained by breeding Crhr1^f/f^ mice to the respective Cre-expressing mouse lines using a three generation breeding scheme. For the selective disruption of CRHR1 in midbrain DAergic neurons, Crhr1^f/f^ mice were bred to DAT-CreERT2 mice. In this transgenic Cre mouse line, an inducible Cre recombinase is controlled by the promoter of the dopamine transporter (DAT) and is preferentially expressed in midbrain dopaminergic neurons (Engblom et al., 2008). The resulting DAT-CreERT2 Crhr1^+/f^ offspring were bred to Crhr1^f/f^ mice, and the line was maintained by breeding DAT-CreERT2 Crhr1^f/f^ mice (DAT-Crhr1) to Crhr1^f/f^ mice. For induction of the mutation, mice were treated with 10 mg of tamoxifen i.p. twice a day for five consecutive days (Eisenhardt et al., 2015) a minimum 6 weeks prior to behavioral experiments. Crhr1^f/f^ control mice were also treated with tamoxifen.

For the selective disruption of CRHR1 in DA receptor 1 positive neurons, Crhr1^f/f^ mice were bred to D1-CreERT2 mice. In this transgenic Cre mouse line, an inducible Cre recombinase is controlled by the promoter of DA receptor 1 and is preferentially expressed in dopaminoceptive neurons (Parkitna et al., 2009; Eisenhardt et al., 2015). D1-CreERT2 Crhr1^f/f^ mice (D1-Crhr1) were obtained by breeding D1-CreERT2 mice to Crhr1^f/f^ mice and maintained as described above. Crhr1^f/f^ mice were backcrossed to C57BL/6N for five generations. For induction of the mutation, mice were treated with 10 mg of tamoxifen i.p. twice a day for five consecutive days (Eisenhardt et al., 2015) a minimum 6 weeks prior to behavioral experiments. Crhr1^f/f^ control mice were also treated with tamoxifen. DAT-CreERT2 and D1-CreERT2 were backcrossed to C57BL/6N for >10 generations before breeding to Crhr1^f/f^ mice.

### Double Fluorescence Immunocytochemistry

Male Crhr1-GFP mice (*n* = 3) were deeply anesthetized with isoflurane and intracardially perfused with 0.9% NaCl containing 10000 IE/1 heparine and subsequently with fixative solution (phosphate buffer containing 4% paraformaldehyde and 14% saturated picric acid). Brains were collected, post-fixed in the same fixative for 1.5 h and transferred to a 10% sucrose solution in 0.1 M phosphate buffer for 48 h (4°C). Brains were frozen in CO_2_ snow and 14 μm coronal cryostat sections were taken at Bregma levels: +1 mm (nucleus accumbens shell, AcbS), +0.1 to +0.02 mm (bed nucleus of the stria terminalis, BNST) and −3 to −3.6 mm (VTA; substantia nigra compacta, SNC; substantia nigra reticulata, SNR, according to the atlas of Paxinos and Franklin (2001). Sections were thaw-mounted on [potassium(III)sulfate · H_2_O] gelatine-coated slides and stored at −20°C. Double fluorescence immunohistochemistry was performed as described by Hansson et al. (2003, 2006b). Briefly, sections were brought to room temperature (RT), rehydrated, rinsed in 0.01 M PBS buffer, and incubated with a mixture of two primary antibodies (rabbit anti-GFP (ThermoFisher Scientific, MA, USA, 1:500) and mouse anti-neuron-specific nuclear protein (NeuN, Merck Millipore, Darmstadt, Germany, 1:700) or sheep anti-tyrosine hydroxylase (TH, Merck Millipore, Darmstadt, Germany, 1:500) or rat anti-D1 (Sigma-Aldrich Chemie, Munich, Germany, 1:800) at 4°C overnight. The sections were rinsed in 0.01 M PBS/0.3% Triton buffer, and a mixture of two secondary antibodies (conjugated with Alexa 488 or Alexa 555: AlexaFluor 488- labeled donkey anti-rabbit, 1:200, and AlexaFluor 555-labeled donkey anti-mouse or AlexaFluor 555-labeled donkey anti-sheep or AlexaFluor 555-labeled donkey anti-rat, 1:100) were added and incubated for 1–2 h at RT. Sections were rinsed briefly, mounted in a mounting medium for fluorescent signals (Dako, Carpinteria, CA, USA), and coverslipped. Sections were then qualitatively analyzed by a Leica TCS SP2 imaging system mounted on a DM IRE2 microscope (Leica Microsystems, Wetzlar, Germany) using an ×63 oil-planchromat lens and either an argon ion laser (458–514 nm) or a green neon laser (543 nm).

### Genotyping and Detection of Conditional Crhr1 Inactivation

Genotyping of conditional Crhr1 knockout mice was performed by PCR as previously described (Kühne et al., 2012). The detection of Crhr1 inactivation in different brain regions was assessed using primers multiplex forward: (a) 5′-AGT-CAA-TCT-GCA-TGT-CCT-CAT-T-3′; and (b) 2cre reverse 5′-ACT-GGG-ACT-AAC-CAT-GGG-TG-3′. Standard PCR conditions using genomic DNA prepared from tissue punches of respective brain regions as template resulted in a 2276 bp PCR product resembling the floxed exon 2 and a 514 bp product following Cre-mediated recombination resembling the inactivated Crhr1 locus lacking exon 2. A wild-type allele would have been detected by a PCR product of 1994 bp. The presence of CreERT2 was assessed by PCR using primers DAT-cre forward 5′-GGC-TGG-TGT-GTC-CAT-CCC-TGA-A-3′ and DAT-cre reverse 5′-GGT-CAA-ATC-CAC-AAA-GCC-TGG-CA-3′. Standard PCR conditions resulted in a 405 bp PCR product.

The site-specific inactivation of Crhr1 was demonstrated by reverse transcriptase PCR (RT-PCR) using cDNA prepared from tissue punches of respective brain areas. The cDNA was used for a first PCR using primers Crhr1-fwd 5′-CTC-TTC-GCT-CTG-GGA-TGT-3′ and Crhr1-rev 5′-CAG-AAA-ACA-ATA-GAA-CAC-AGA-CAC-3′. This PCR product was used as template for subsequent nested PCRs. The wild-type Crhr1 transcript was detected by nested PCR using primers nested CRHR1 forward (c) 5′-GAG-CCC-GAG-GAT-GGG-ACA-GC-3′ and Crhr1-E5-rev (d) 5′-TGA-CGG-CAA-TGT-GGT-AGT-GC-3′as a PCR product of 371 bp. The mutant Crhr1 transcript following Cre recombinase-mediated deletion of exon 2 was detected by nested PCR using primers Crhr1-E1-3-fwd2 (e) 5′-CTC-CGG-CTC-GTG-AAG-GCC-TG-3′ and Crhr1-E5-rev (d) as a 255 bp PCR product.

### Drugs

Cocaine hydrochloride (Sigma-Aldrich, Germany) was dissolved in physiological saline (0.9% NaCl) for intraperitoneal (IP) injection of 15 mg/kg (10 ml/kg) for locomotor activity measurements or 0.50 mg/kg/14 μl infusion for self-administration.

Tamoxifen was dissolved in medium-chain triglycerides (Stadtklinik Frankenthal, Frankenthal, Germany) to a final concentration of 10 mg/ml.

### Behavioral Procedures

#### Locomotor Activity

The acute and sensitized locomotor response to cocaine was assessed in eight TruScan activity monitors (Coulbourn Instruments, USA). Each monitor consists of a clear acrylic plastic test cage (27 × 27 × 39 cm) placed inside a monitoring unit that records via computer ambulatory beam interruptions from infrared photocell emitter/detector pairs evenly spaced along each axis. Mice were habituated to the locomotor activity monitors for 60 min/day for three consecutive days. Following habituation, mice received 15 mg/kg cocaine IP or saline (10 ml/kg) on subsequent days for 5 days (five injections). Cocaine injections on days 1 and 5 were followed by 60-min locomotor measurements in the activity monitors. Locomotor activity was measured as distance traveled (cm).

#### Cocaine Self-Administration

Self-administration was assessed in 12 operant chambers (Med Associates, USA) housed in light- and sound-attenuating cubicles. Each chamber (24.1 × 20.3 × 18.4 cm) is equipped with two levers (left and right), a food dispenser and a drug delivery system connected via infusion pump (PHM-100, Med-Associates, USA) located outside the cubicle. Operant chambers were controlled using Med-PC IV (Med Associates, USA) software. Mice first underwent lever training with 14 mg sweetened food pellets (TestDiet, USA), as previously described (Bernardi and Spanagel, 2013). Following lever training, mice were implanted with an indwelling intravenous catheter (made in-house) into the jugular vein. Catheter patency was maintained with 0.15 ml heparanized saline (100 i.u./ml) containing Baytril (0.7 mg/ml) administered daily throughout the experiment. After 3 days recovery, mice underwent daily 2 h cocaine self-administration for eight consecutive days. Cocaine (0.50 mg/kg/14 μl infusion) delivery was contingent upon pressing on the active lever under an FR1 schedule of reinforcement and paired with the 20 s presentation of a blinking light stimulus (Conditioned Stimulus, CS), which also served as a timeout period, during which lever presses were not reinforced. For all experiments, presses on the inactive lever were recorded but had no scheduled consequence.

Following cocaine self-administration, mice were exposed to daily 2 h extinction sessions, during which presses on the active lever were not reinforced, and the CS was omitted. Extinction concluded when each mouse reached a criterion level of responding on the active lever (less than 33% responding over 2 days relative to the mean active lever presses during the last 2 days of self-administration). Once extinction criteria were reached, cue reinstatement was tested during a single 2 h session that began with a 20 s blinking of the light CS, and during which active lever presses resulted in the presentation of the 20 s blinking light CS, but no cocaine. Following cue reinstatement testing, further tests for yohimbine-induced reinstatement and yohimbine + cue reinstatement were conducted, and are described in *Supplementary Information*.

### Statistical Analysis

Statistical analyses were conducted using SPSS software (StatSoft, USA). Locomotor data were analyzed using three-way ANOVA (treatment × day × genotype). SA acquisition data were analyzed using three-way ANOVA (lever × day × genotype), while extinction and reinstatement data were analyzed using three-way (session × lever × genotype) and two-way ANOVA (lever × genotype). *Post hoc* confirmation of effects was conducted using Bonferroni-corrected *t*-tests, where indicated. Significance was set at *p* < 0.05.

## Results

### Co-Localization of CRHR1 in Dopaminergic and D1-Expressing Neurons

**Figure 1:**
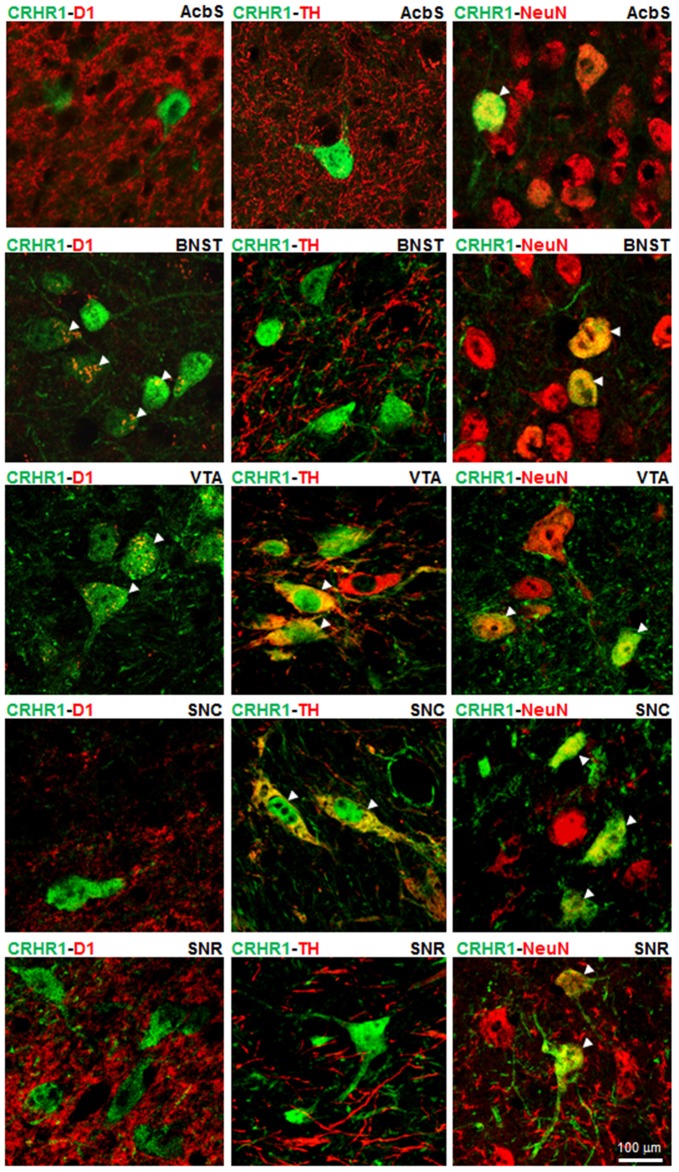
Corticotropin-releasing hormone receptor subtype 1 (CRHR1) is expressed in both DAergic and D1 neurons. Double fluorescence immunohistochemistry for (Crhr1-)GFP-ir (green) and tyrosine hydroxylase (TH)-ir (red) or D1-ir (red) or NeuN-ir (red) on coronal brain sections of Crhr1-GFP reporter mice (Justice et al., 2008) in reward-related brain regions (Noori et al., 2012). Merged images show the expression of (Crhr1-)GFP-ir (green) in DAergic TH-ir positive cells (red) in the ventral tegmental area (VTA) and the substantia nigra compacta (SNC). A co-localization of (Crhr1-)GFP-ir (green) and D1-ir (red) is detected in the bed nucleus of the stria terminalis (BNST) and the VTA. White arrow heads point to co-localized CRHR1 with either TH-ir or D1-ir cells (yellow color). Co-localized CRHR1 with TH-ir and D1-ir is not found in the accumbens shell (AcbS) or substantia nigra reticulata (SNR). Double immunostainings for (Crhr1-)GFP-ir (green) and NeuN-ir (red) demonstrates a neuronal location of CRHR1 in all analyzed brain regions.

(Crhr1-)GFP-ir and NeuN-ir were primarily found in the nuclei of neurons with lighter staining in the cytoplasm, while TH-ir was detected exclusively in the cytoplasm and D1-ir on neuronal dendrites. Double fluorescence immunolabelings of (Crhr1-) GFP-ir with NeuN-ir demonstrated a neuronal location of CRHR1 in all analyzed reward-related brain regions (i.e., AcbS, BNST, VTA, SNC, SNR; Noori et al., 2012). (Crhr1-)GFP-ir was expressed in TH-ir positive DAergic neurons of the VTA and the SNC, while (Crhr1-)GFP-ir in D1-ir neurons was detected in the BNST and the VTA. In the AcbS and SNR, (Crhr1-)GFP-ir was not found in either DAergic TH-ir or D1-ir expressing neurons. All immunostainings are shown in Figure 1 and Table 1. In summary, we demonstrate that CRHR1 is expressed in both DAergic and D1 dopaminoceptive neurons.

**Table 1:** Localization of corticotropin-releasing hormone receptor subtype 1 (CRHR1) in tyrosine hydroxylase (TH) and D1 expressing cells.

	CRHR1-D1	CRHR1-TH	CRHR1-NeuN
AcbS	−	−	+++
BNST	++	−	+++
VTA	++	++	+++
SNC	−	++	++
SNR	−	−	++

*Nucleus accumbens shell (AcbS); bed nucleus of the stria terminalis (BNST); ventral tegmental area (VTA); substantia nigra compacta (SNC); substantia nigra reticulata (SNR), no (−), sparse (+), modest (++), high (+++) level of co-localized (Crhr1-)GFP-ir with either D1-ir, TH-ir or NeuN-ir. Co-localization was performed in (Crhr1-)GFP reporter mice (*n* = 3, Justice et al., 2008)*.

### Generation of Conditional Knockouts

**Figure 2:**
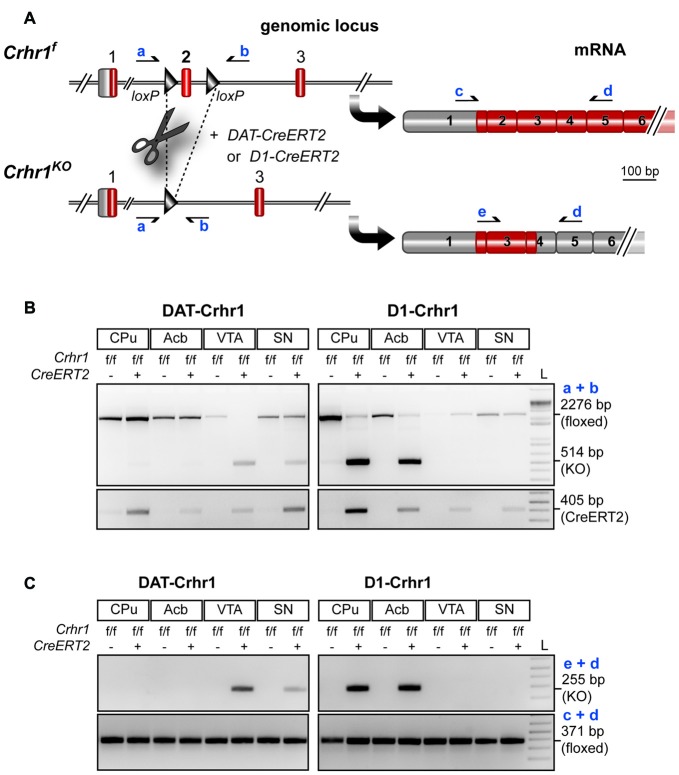
Conditional inactivation of Crhr1 in dopamine transporter (DAT)-Crhr1 and D1-Crhr1 mice. **(A)** Schematic illustration of conditional Crhr1 allele (Crhr1^f^) in which the critical exon 2 is flanked by loxP sites and corresponding mRNA transcripts. Cre recombinase-mediated deletion of exon 2 results in functional inactivation of Crhr1 (Crhr1^KO^) due to a premature stop codon in exon 4 Primers (a) and (b) for detection of the recombination event on genomic level as well as primers (c), (d) and (e) for detection of the mutant transcript are depicted. **(B)** Brain region-specific detection of Crhr1 inactivation on the genomic level in dopaminergic neurons in the VTA and substantia nigra (SN) of DAT-Crhr1 mice as well as in dopaminoceptive neurons in the caudate putamen (CPu) and nucleus accumbens (Acb) of D1-Crhr1 mice. The floxed exon 2 and thus intact Crhr1 is detected by a 2276 bp PCR product. Deletion of exon 2 and concomitant inactivation of Crhr1 is detected by a 514 bp PCR product. Conditional Crhr1 inactivation is based on the presence of tamoxifen-inducible CreERT2 detected by a 405 bp PCR product. **(C)** Brain region-specific detection of the mutant Crhr1 transcript lacking exon 2 in the VTA and SN of DAT-Crhr1 and CPu and Acb of D1-Crhr1 mice. The Crh1 transcript lacking exon 2 is detected by a 255 bp PCR product. As a control the wild-type transcript was detected in all brain regions analyzed in DAT-Crhr1 and D1-Crhr1 mice. Wild-type Crhr1 transcripts present in regions of Crhr1 deletion originate from Crhr1 expressed in non-dopaminergic or non-dopaminoceptive cells, respectively. Translated exons are depicted in red. 5′UTR and untranslated exons are depicted in gray.

In Figure 2A, a schematic illustration of the conditional Crhr1 allele (Crhr1^fl^) in which the critical exon 2, flanked by loxP sites, is depicted. Conditional Crhr1 inactivation is based on the presence of tamoxifen-inducible CreERT2, and Cre recombinase-mediated deletion of exon 2 resulted in functional inactivation of Crhr1 (Crhr1^KO^). Brain region-specific detection of Crhr1 inactivation in DAergic neurons in the VTA and SN of DAT-Crhr1 mice, as well as in dopaminoceptive neurons in the caudate putamen (CPu) and Acb of D1-Crhr1 mice, was demonstrated on genomic and mRNA level as depicted in Figures 2B,C, respectively.

### Behavior

#### Locomotor Activity

**Figure 3:**
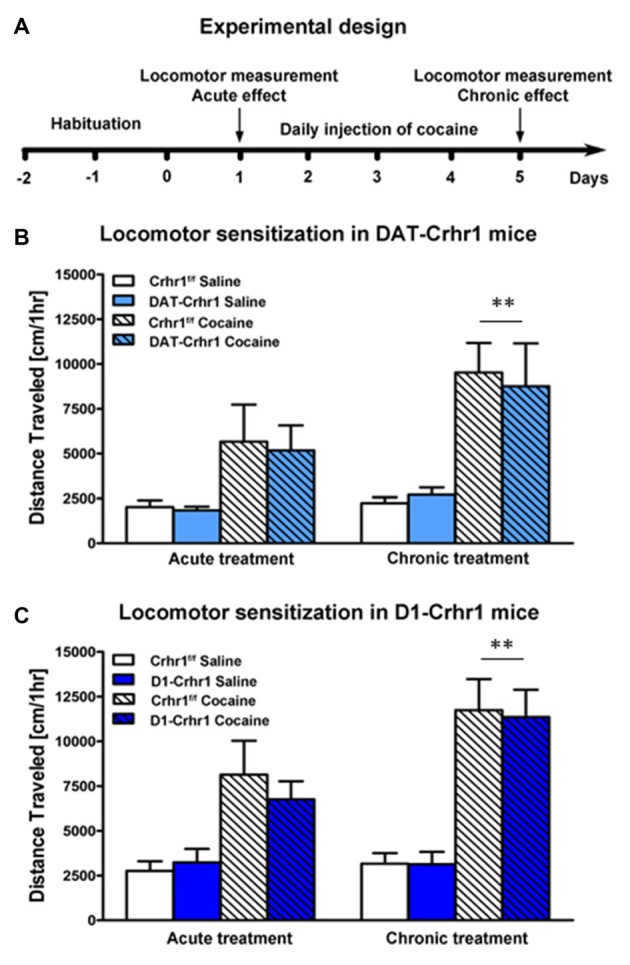
Acute and sensitized locomotor response to cocaine in DAT-Crhr1 and D1-Crhr1 mice. **(A)** Experimental design for cocaine locomotor sensitization. There were no differences in the acute or locomotor response to cocaine in **(B)** DAT-Crhr1 and **(C)** D1-Crhr1 mice and their respective littermate Crhr1^f/f^ controls. Furthermore, saline-treated groups revealed no differences in basal locomotor activity among the groups. Data represent mean activity (±SEM) during the 60-min locomotor activity trial on Day 1 and Day 5 of cocaine or saline exposure. **p < 0.005 across genotypes relative to Day 1.

DAT-Crhr1 mice and littermate Crhr1^f/f^ control mice did not differ in the locomotor response to saline (*n* = 7 and *n* = 9 for DAT-Crhr1 and control mice, respectively) or the acute and sensitized locomotor response to cocaine (*n* = 7 and *n* = 8 for DAT-Crhr1 and control mice, respectively). Figure 3A shows the experimental design for locomotor testing. Figure 3B shows the locomotor response to saline or cocaine on Days 1 and 5 for each group. Data represent mean (±SEM) distance traveled (cm) across the 60-min activity trials. A three-way ANOVA (treatment × day × genotype) revealed significant main effects of both treatment (*F*_(1,27)_ = 15.7, *p* < 0.005) and day (*F*_(1,27)_ = 29.7, *p* < 0.001) and a significant treatment × day interaction (*F*_(1,27)_ = 16.7, *p* < 0.001), but no other effects (*F*s < 1). Paired *t*-tests analyzing cocaine-treated animals confirmed that across genotypes there was a significant increase in locomotion from Day 1 to Day 5 (*t*_(14)_ = 5.1, *p* < 0.005, Bonferroni-corrected *α* = 0.05/2), confirming sensitization. Saline-treated animals (*t*_(15)_ = 2.0, *p* > 0.025, Bonferroni-corrected *α* = 0.05/2) demonstrated no such increase. These results indicate no differences in basal locomotor activity, or the acute and sensitized response to cocaine, as a function of genotype.

D1-Crhr1 and littermate Crhr1^f/f^ control mice did not differ in the locomotor response to saline (*n* = 9 and *n* = 10 for D1-Crhr1 and control mice, respectively) or the acute and sensitized locomotor response to cocaine (*n* = 9 and *n* = 10 for D1-Crhr1 and control mice). Figure 3C shows the locomotor response to saline or cocaine on Days 1 and 5 for each group. Data represent mean (±SEM) distance traveled (cm) across the 60-min activity trials. A three-way ANOVA (treatment × day × genotype) revealed significant main effects of both treatment (*F*_(1,34)_ = 29.1, *p* < 0.001) and day (*F*_(1,34)_ = 48.2, *p* < 0.001) and a significant treatment × day interaction (*F*_(1,34)_ = 41.7, *p* < 0.001), but no other effects (*F*s < 1, except treatment × day × genotype: *F*_(1,34)_ = 1.5, *p* > 0.05). Paired *t*-tests analyzing cocaine-treated animals confirmed that across genotypes there was a significant increase in locomotion from Day 1 to Day 5 (*t*_(18)_ = 7.3, *p* < 0.005, Bonferroni-corrected *α* = 0.05/2), confirming sensitization. Saline-treated animals (*t*_(18)_ = 0.7, *p* > 0.025, Bonferroni-corrected *α* = 0.05/2) demonstrated no such increase. These results indicate no differences in basal locomotor activity, or the acute and sensitized response to cocaine, as a function of genotype.

#### Cocaine Self-Administration

**Figure 4:**
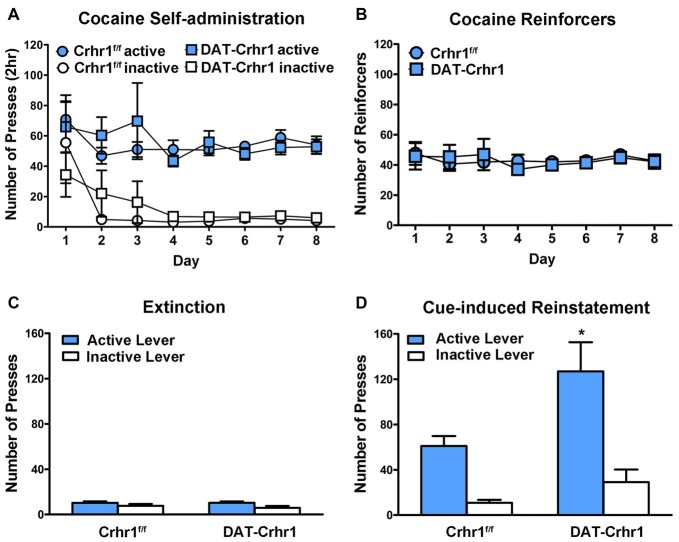
Cocaine self-administration in DAT-Crhr1 and littermate Crhr1^f/f^ control mice. **(A)** Both DAT-Crhr1 and controls demonstrated similar acquisition of the task, as demonstrated by a significant difference between responding on the active and inactive levers. Data represent mean number of presses (±SEM) on the active and inactive levers during eight daily 2 h sessions of cocaine self-administration (0.50 mg/kg/infusion). **(B)** There was no difference in DAT-Crhr1 mice and controls in the number of cocaine reinforcers achieved. Data represent mean number of cocaine reinforcers (±SEM) achieved during eight daily 2 h sessions of cocaine self-administration (0.50 mg/kg/infusion). **(C)** There was no difference in responding in DAT-Crhr1 mice and controls during the final 2 days of extinction trials. Data represent mean number of presses (±SEM) on the active and inactive levers during the final 2 days of daily 2 h extinction sessions. **(D)** DAT-Crhr1 mice demonstrated increased cue-induced reinstatement relative to controls, as indicated by an increase in responding on the active lever without a corresponding change in responding on the inactive lever. Data represent mean number of presses (±SEM) on the active and inactive levers during a single 2 h session of cue reinstatement. *p < 0.025 relative to active lever presses of control mice.

DAT-Crhr1 (*n* = 7) and littermate Crhr1^f/f^ control (*n* = 8) mice did not differ in cocaine self-administration. Figure 4A shows the mean (±SEM) responding on the active and inactive levers during eight daily 2 h sessions of cocaine self-administration. A three-way ANOVA (lever × day × genotype) revealed significant main effects of lever (*F*_(1,13)_ = 221.1, *p* < 0.001), indicating a distinction between the active and inactive levers, and a significant main effect of day (*F*_(2.0,25.6)_ = 3.4, *p* < 0.05). However, there was no main effect of genotype and no interactions (*F*s < 1, except day × lever: *F*_(2.0,25.6)_ = 1.8, *p* > 0.05). Figure 4B shows the mean (±SEM) number of cocaine infusions received during 8 days of cocaine self-administration. A two-way ANOVA (day × genotype) revealed no significant effects (*F*s < 1), indicating no difference in reinforcers based on genotype.

Following the self-administration procedure, DAT-Crhr1 mice and controls underwent extinction and subsequent reinstatement testing, during which all mice demonstrated a return to responding on the cocaine-associated lever. A three-way ANOVA (session × lever × genotype) revealed a significant session × lever × genotype interaction (*F*_(1,13)_ = 5.3, *p* < 0.05), significant session × genotype (*F*_(1,13)_ = 6.7, *p* < 0.05), lever × genotype (*F*_(1,13)_ = 5.7, *p* < 0.05) and session × lever (*F*_(1,13)_ = 50.0, *p* < 0.001) interactions, and significant main effects of session (*F*_(1,13)_ = 34.0, *p* < 0.001), lever (*F*_(1,13)_ = 56.1, *p* < 0.001), and genotype (*F*_(1,13)_ = 5.5, *p* < 0.05). Importantly, paired samples *t*-tests of the active and inactive levers between extinction and reinstatement revealed that active presses increased in both DAT-Crhr1 mice (*t*_(6)_ = 4.6, *p* < 0.005, Bonferroni-corrected *α* = 0.05/4 = 0.0125) and controls (*t*_(7)_ = 6.0, *p* < 0.005, Bonferroni-corrected *α* = 0.05/4 = 0.0125). Inactive lever presses did not differ across sessions in DAT-Crhr1 mice (*t*_(6)_ = 2.1, *p* > 0.0125, Bonferroni-corrected *α* = 0.05/4) and controls (*t*_(7)_ = 1.2, *p* > 0.0125, Bonferroni-corrected *α* = 0.05/4). Furthermore, responding at the conclusion of extinction did not differ between DAT-Crhr1 mice and controls. Figure 4C shows the mean (±SEM) responding on the active and inactive levers during the final 2 days of 2 h daily extinction sessions. A two-way ANOVA (lever × genotype) revealed a significant main effect of lever (*F*_(1,13)_ = 21.9, *p* < 0.001), but no main effect of genotype (*F* < 1) and no interaction (*F*_(1,13)_ = 1.6, *p* > 0.05). Furthermore, DAT-Crhr1 mice and controls did not differ in the number of trials required to reach the extinction criteria (*t*_(13)_ = 1.2, *p* > 0.05; for DAT-Crhr1 mice and controls, 11.6 ± 4.0 and 6.8 ± 1.5 extinction sessions, respectively). During cue reinstatement, DAT-Crhr1 mice demonstrated higher levels of responding on the cocaine-associated active lever relative to controls. Figure 4D shows the mean (±SEM) responding on the active and inactive levers during the 2-h cue reinstatement test. A two-way ANOVA (lever × genotype) revealed main effects of genotype (*F*_(1,13)_ = 6.1, *p* < 0.05) and lever (*F*_(1,13)_ = 53.3, *p* < 0.001), but more importantly, a significant lever × genotype interaction (*F*_(1,13)_ = 5.5, *p* < 0.05). Independent samples *t*-tests confirmed that DAT-Crhr1 mice responded more on the active lever than control mice (*t*_(13)_ = 2.6, *p* < 0.025, Bonferroni-corrected *α* = 0.05/2), but the two groups did not differ on inactive lever pressing (*t*_(13)_ = 1.8, *p* > 0.025, Bonferroni-corrected *α* = 0.05/2), indicating a selective increase in responding on the cocaine-associated lever by DAT-Crhr1 mice relative to controls. For yohimbine-induced reinstatement and yohimbine + cue reinstatement in DAT-Crhr1 mice, please see Supplementary Figure S1 (*Supplementary Information*).

**Figure 5:**
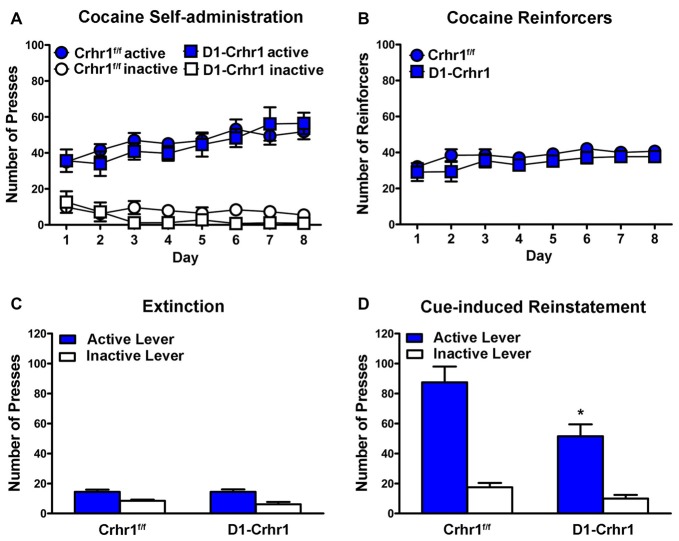
Cocaine self-administration in D1-Crhr1 and littermate Crhr1^f/f^ control mice. **(A)** Both D1-Crhr1 and controls demonstrated similar acquisition of the task, as demonstrated by a significant difference between responding on the active and inactive levers. Data represent mean number of presses (±SEM) on the active and inactive levers during eight daily 2 h sessions of cocaine self-administration (0.50 mg/kg/infusion). **(B)** There was no difference in D1-Crhr1 mice and controls in the number of cocaine reinforcers achieved. Data represent mean number of cocaine reinforcers (±SEM) achieved during eight daily 2 h sessions of cocaine self-administration (0.50 mg/kg/infusion). **(C)** There was no difference in responding in D1-Crhr1 mice and controls during the final 2 days of extinction trials. Data represent mean number of presses (±SEM) on the active and inactive levers during the final 2 days of daily 2 h extinction sessions. **(D)** D1-Crhr1 mice demonstrated decreased cue-induced reinstatement relative to controls, as indicated by a decrease in responding on the active lever without a corresponding change in responding on the inactive lever. Data represent mean number of presses (±SEM) on the active and inactive levers during a single 2 h session of cue reinstatement. *p < 0.025 relative to active lever presses of control mice.

D1-Crhr1 (*n* = 9) and littermate Crhr1^f/f^ control (*n* = 10) mice did not differ in cocaine self-administration. Figure 5A shows the mean (±SEM) responding on the active and inactive levers during eight daily 2 h sessions of cocaine self-administration. A three-way ANOVA (lever × day × genotype) revealed a significant main effects of lever (*F*_(1,17)_ = 140.6, *p* < 0.001), indicating a distinction between the active and inactive levers, and a significant day × lever interaction (*F*_(4.1,69.8)_ = 9.5, *p* < 0.001). However, there was no main effect of genotype (*F*_(1,17)_ = 1.4, *p* > 0.05) or genotype × lever interaction (*F* < 1), and no other main effects or interactions (day: *F*_(3.5,58.8)_ = 2.3, *p* > 0.05; day × genotype: *F*_(3.5,58.8)_ = 1.0, *p* > 0.05; day × lever × genotype: *F*_(4.1,69.8)_ = 1.2, *p* > 0.05). Figure 5B shows the mean (±SEM) number of cocaine infusions received during 8 days of cocaine self-administration. A two-way ANOVA (day × genotype) revealed a significant main effect of day (*F*_(3.7,62.4)_ = 3.7, *p* < 0.05), but no significant main effect of genotype (*F*_(1,17)_ = 1.5, *p* > 0.05) and no day × genotype interaction (*F* < 1), indicating no difference in reinforcers based on genotype.

Following the self-administration procedure, D1-Crhr1 mice and controls underwent extinction and subsequent reinstatement testing, during which all mice demonstrated a return to responding on the cocaine-associated lever. A three-way ANOVA (session × lever × genotype) revealed a significant session × lever × genotype interaction (*F*_(1,17)_ = 5.9, *p* < 0.05), significant session × genotype (*F*_(1,17)_ = 9.8, *p* < 0.05) and session × lever (*F*_(1,17)_ = 59.6, *p* < 0.001) interactions (lever × genotype interaction: *F*_(1,17)_ = 3.7, *p* > 0.05), and significant main effects of session (*F*_(1,17)_ = 88.0, *p* < 0.001), lever (*F*_(1,17)_ = 86.5, *p* < 0.001), and genotype (*F*_(1,17)_ = 7.3, *p* < 0.05). Importantly, paired samples *t*-tests of the active and inactive levers between extinction and reinstatement revealed that active presses increased in both D1-Crhr1 mice (*t*_(8)_ = 5.0, *p* < 0.005, Bonferroni-corrected *α* = 0.05/4 = 0.0125) and controls (*t*_(9)_ = 7.6, *p* < 0.001, Bonferroni-corrected *α* = 0.05/4 = 0.0125). Inactive lever presses did not differ across session in D1-Crhr1 mice (*t*_(8)_ = 1.9, *p* > 0.0125, Bonferroni-corrected *α* = 0.05/4), but differed slightly in controls (*t*_(9)_ = 3.4, *p* < 0.0125, Bonferroni-corrected *α* = 0.05/4). Furthermore, responding at the conclusion of extinction did not differ between D1-Crhr1 mice and controls. Figure 5C shows the mean (±SEM) responding on the active and inactive levers during the final 2 days of 2 h daily extinction sessions. A two-way ANOVA (lever × genotype) revealed a significant main effect of lever (*F*_(1,17)_ = 43.0, *p* < 0.001), but no main effect of genotype (*F*s < 1) and no interaction (*F*_(1,17)_ = 1.1, *p* > 0.05). Furthermore, D1-Crhr1 mice and controls did not differ in the number of trials required to reach the extinction criteria (*t*_(17)_ = 0.1, *p* > 0.05; for D1-Crhr1 mice and controls, 8.2 ± 3.3 and 8.6 ± 1.3 extinction sessions, respectively). During cue reinstatement, D1-Crhr1 mice demonstrated lower levels of responding on the cocaine-associated active lever relative to controls. Figure 5D shows the mean (±SEM) responding on the active and inactive levers during the 2-h cue reinstatement test. A two-way ANOVA (lever × genotype) revealed main effects of genotype (*F*_(1,17)_ = 8.6, *p* < 0.01) and lever (*F*_(1,17)_ = 74.4, *p* < 0.001), but more importantly, a significant lever × genotype interaction (*F*_(1,17)_ = 4.8, *p* < 0.05). Independent samples *t-tests* confirmed that D1-Crhr1 mice responded less on the active lever than control mice (*t*_(17)_ = 2.7, *p* < 0.025, Bonferroni-corrected *α* = 0.05/2), but the two groups did not differ on inactive lever pressing (*t*_(17)_ = 2.0, *p* > 0.025, Bonferroni-corrected *α* = 0.05/2), indicating a selective decrease in responding on the cocaine-associated lever by D1-Crhr1 mice relative to controls. For yohimbine-induced reinstatement and yohimbine + cue reinstatement in D1-Crhr1 mice, please see Supplementary Figure S2 (*Supplementary Information*).

Because of the differences in active lever responding during the reinstatement tests for the Crhr1^f/f^ control mice of the Crhr1-DAT and Crhr1-D1 lines, we further analyzed these levels of responding relative to final extinction values (the mean of the last two extinction days), finding that these percentage differences did not differ between the lines. Supplementary Figure S3 (*Supplementary Information*) shows the percentage (±SEM) responding during cue reinstatement relative to the mean of the final 2 days of 2 h daily extinction sessions on the active lever for both Crhr1-DAT and Crhr1-D1 mice and their respective controls. A two-way ANOVA of active lever presses (line × genotype) revealed main effects of line (*F*_(1,30)_ = 15.3, *p* < 0.001) and a significant line × genotype interaction (*F*_(1,30)_ = 11.6, *p* < 0.005), but no effect of genotype (*F*_(1,30)_ = 2.0, *p* > 0.05). Importantly, there was no difference in percentage responding relative to final extinction values in the two control lines (*t*_(16)_ = 0.4, *p* > 0.05); thus, although absolute values of responding on the active lever differed between the Crhr1^f/f^ control mice of the Crhr1-DAT and Crhr1-D1 lines, the levels of responding relative to extinction did not differ.

## Discussion

Here we report dissociable effects of CRHR1 inactivation in DAT- and D1-containing neurons on cue-induced reinstatement following cocaine self-administration. DAT-Crhr1 mice demonstrated a significant increase in cue-induced reinstatement relative to controls, while D1-Crhr1 mice demonstrated a significant decrease in cue-induced reinstatement relative to controls, indicating neuronal selectivity of CRHR1 receptors in the control of relapse-like behavior. Furthermore, we demonstrated that CRHR1 in DAT and D1-containing neurons are not involved in mediating the primary rewarding properties of cocaine, consistent with previous studies that have used both pharmacological and viral approaches to examine the role of CRHR1 on self-administration specifically under limited access conditions (Przegalinski et al., 2005; Chen et al., 2014), and had no effect on basal locomotion or the acute and sensitized locomotor response to cocaine. These findings suggest that CRHR1 may play an important role in cue-induced reinstatement based on neuronal localization. Functional *in vivo* experiments have previously revealed bidirectional models for the role of CRHR1 on the cellular level in anxiety studies (Refojo et al., 2011). Thus, an imbalance between CRHR1-controlled drug-seeking responses in DAergic and D1-containing dopaminoceptive neurons may lead to a systemic craving response. Nonetheless, in addition to a role for CRHR1 in stress-induced reinstatement, our findings further confirm the involvement of CRHR1 receptors in cue-induced cocaine-seeking.

Our findings in D1-Crhr1 mice, which showed a decreased cue-induced reinstatement relative to controls, are consistent with previous work demonstrating an attenuation of drug-seeking in various reinstatement studies using a variety of drugs of abuse by peripheral administration of CRHR1 antagonists following drug self-administration (e.g., Shaham et al., 1998; Goeders and Clampitt, 2002; Przegalinski et al., 2005; Marinelli et al., 2007; Zislis et al., 2007; Smith and Aston-Jones, 2011; Cosme et al., 2015). Few studies to date have specifically examined the effect of CRHR1 antagonism on cocaine *cue*-mediated reinstatement following extinction, similar to the paradigm used here. Peripheral administration of a CRHR1 antagonist resulted in a decrease in cue-induced cocaine-seeking (Goeders and Clampitt, 2002; Smith and Aston-Jones, 2011), an effect also demonstrated with a CRHR1 antagonist administered into the insular cortex (Cosme et al., 2015). However, in addition, intracerebroventricular CRHR1 antagonism has been shown to attenuate methamphetamine cue-induced reinstatement (Moffett and Goeders, 2007).

Because our inducible line specifically knocked out CRHR1 in D1-containing neurons, and D1 receptors are expressed in low levels in the VTA, as indicated by our immunostainings (see also Bernardi et al., 2015), we suggest that conditioned cue-induced drug-seeking is at least partly mediated by CRHR1 in DA projection regions demonstrated to play a role in cocaine cue-induced reinstatement (Kalivas and McFarland, 2003; Kalivas and Volkow, 2005; See, 2005). Reinstatement to drug-seeking requires D1 activation in the PFC and basolateral amygdala (BLA; Ciccocioppo et al., 2001; Kruzich et al., 2001; Capriles et al., 2003), which mediate excitatory drive and associative processes related to drug-mediated behaviors (Everitt et al., 1999; Peters et al., 2009). CRH acting via CRHR1 has been demonstrated to potentiate D1 receptor modulation of a BLA-PFC pathway (Orozco-Cabal et al., 2008). Furthermore, CRHR1 is required for D1-mediated signaling in the BNST (Kash et al., 2008), part of the extended amygdala that also subserves drug-seeking behaviors (Koob and Le Moal, 2008a); importantly, projections from the BNST to the VTA comprise an important substrate mediating the actions of CRH and CRHR1 on stress-induced drug-seeking behaviors (reviewed in Silberman and Winder, 2013) and clear co-localization of Crhr1 and D1 in the BNST can be seen in our immunostainings. In addition, systemic pretreatment with the D1 antagonist SCH23390 blocked intracerberoventicular CRF-induced reinstatement of cocaine seeking (Brown et al., 2012). Thus, CRHR1 plays a modulatory role in D1-mediated signaling. The loss of CRHR1 in D1-expressing neurons in the BLA and/or PFC, as well as in the extended amygdala and in BLA-CeA projections that may also process stimulus-reward associations (Pollandt et al., 2006; Krishnan et al., 2010), likely underlie the decreased cue-induced reinstatement in D1-Crhr1 mice demonstrated in the current study.

CRHR1 on DAT-containing neurons are likely predominantly located on cell bodies and dendrites in the VTA (see our immunostainings), implicating the loss of CRHR1 in the VTA in our results in DAT-Crhr1 mice. Our demonstration of an increase in cue-mediated drug-seeking in DAT-Crhr1 mice relative to controls is not entirely consistent with previous findings, which have generally implicated an inhibitory role of CRHR1 antagonists in the VTA on reinstatement under various protocols (Blacktop et al., 2011; Vranjkovic et al., 2014; but see, e.g., Wang et al., 2007), including a recent report that demonstrated a decrease in cue-mediated drug-seeking in mice relative to controls following lentiviral knockdown of CRHR1 in the VTA (Chen et al., 2014). However, it is important to note that CRHR1 in the VTA are located both presynaptically and postsynaptically, as indicated by our immunostainings, primarily located on glutamatergic and dopaminergic neurons (Sauvage and Steckler, 2001; Hahn et al., 2009; Refojo et al., 2011; Williams et al., 2014). Lentiviral targeting of CRHR1 in the VTA may have resulted in alterations in signaling in glutamatergic neurons expressing CRHR1 in addition to CRHR1-containing DAergic neurons. In fact, cocaine experience has been demonstrated to result in a sustained enhancement of CRH potentiation of glutamate transmission onto DA neurons in the VTA (Wang et al., 2005; Hahn et al., 2009; Williams et al., 2014), such that lentiviral knockdown—as well as pharmacological inhibition—of CRHR1 on both glutamatergic and DAergic neurons in the VTA may result in a complete inhibition of CRHR1-mediated excitability mediating drug-seeking. In contrast, our inducible knockdown is specific to DAergic neurons. The mechanism by which these differences might differentially effect cue-mediated reinstatement remains unclear, but may involve an opposing, but concerted action of CRHR1 on glutamate and DA neurons in the VTA in the regulation of behavior (Refojo et al., 2011). Nonetheless, our findings suggest that the role of CRHR1 may be much more complex than previously demonstrated. In fact, a recent study showed that mice globally deficient in CRHR1 showed a leftward shift of the dose response curve for cocaine reward—in other words, an increase in sensitivity at lower doses—as measured by conditioned place preference (Contarino et al., 2017), again confirming somewhat divergent effects of CRHR1 knockdown or inhibition on reward processes.

Other factors may also contribute to the increase in reinstatement demonstrated in Crhr1-DAT mice. CRHR1 on nerve terminals in the Acb, despite our difficulty in characterizing CRHR1 on DAT-containing (as well as D1) neurons in this region, may also play a role in our observed effect, as CRHR1 antagonist administered directly into the Acb have shown involvement in drug reinstatement (Wang et al., 2006). CRHR1 on nerve terminals in the PFC (Sauvage and Steckler, 2001) may also play a role in the effects seen in Crhr1-DAT mice. In fact, CRHR1 antagonists have been demonstrated to increase cocaine-induced DA overflow in the PFC (Gurkovskaya et al., 2005), an area critical for cue-induced reinstatement (Ciccocioppo et al., 2001; McLaughlin and See, 2003). Furthermore, the lack of CRHR1 on dopaminergic neurons may result in an increase in the extracellular concentration of CRH, which may be recruited by CRH binding protein (CRH-BP). CRH-BP activates CRHR2 in the VTA, facilitating calcium flow via an NMDA-mediated pathway and in turn resulting in an increase in burst firing of DA neurons and glutamate and DA levels (Ungless et al., 2003; Wang et al., 2007). CRHR2 also regulates glutamate release onto VTA DA neurons via disinhibition of the GABAergic suppression of glutamatergic cells (Williams et al., 2014). Together, these studies suggest the possibility of a compensatory mechanism of increased DA release that may explain the increased reinstatement in DAT-Crhr1 mice. The concerted actions between pre- and postsynaptic CRHR1 in the VTA, as well as the role of CRHR2, which play an equally important role in VTA excitability (Hahn et al., 2009; Williams et al., 2014) and drug-seeking behaviors (Wang et al., 2007; Guan et al., 2014), need further evaluation in terms of conditioned reinforcement. Nonetheless, our findings and those previously reported indicate an important role of CRHR1 on drug-seeking behaviors.

Interestingly, yohimbine alone had only a minor, but significant, effect on responding in the two lines, but there were no differences between the genotypes (*Supplementary Information*). Yohimbine has previously been used as a stressor to induce reinstatement of cocaine conditioned place preference in mice (Mantsch et al., 2010; Sartor et al., 2016), but has to date not been reported to induce reinstatement of drug-seeking following cocaine self-administration and extinction in mice. However, it has effectively been used to induce reinstatement of cocaine-seeking in rats (e.g., Feltenstein and See, 2006; Feltenstein et al., 2011; Brown et al., 2012). Thus, yohimbine may be more effective in rats at doses similar to that used here; higher doses may be necessary to produce a more robust reinstatement of cocaine-seeking in mice. Furthermore, a more conventional stressor, such as footshock, may have more reliably induced stress-related reinstatement in mice. Indeed, systemic yohimbine has been shown to increase impulsivity (Mahoney et al., 2016; Adams et al., 2017) that may affect behaviors such as lever responding. Nonetheless, it is difficult to conclude here that yohimbine resulted in a reliable stress-induced reinstatement, and further studies are required to assess the effectiveness of yohimbine in self-administration in mice. Importantly, however, our subsequent yohimbine + cue reinstatement testing replicated our cue-induced reinstatement findings (*Supplementary Information*), and as yohimbine appeared to play only a minor role on its own, it is likely reinstatement responding was almost entirely due to the presence of the cue, confirming the bidirectional effect of DAT-Crhr1 and D1-Crhr1 knockdown on cue-mediated relapse-like responding. It is important to mention that although the number of days to criterion during the first bout of extinction sessions prior to cue reinstatement did not differ significantly relative to controls in either of the lines, there were significant differences in these values prior to yohimbine and yohimbine + cue reinstatement, with DAT-Crhr1 and D1-Crhr1 showing impaired and accelerated extinction training, respectively, relative to the appropriate control groups. Because tamoxifen was administered prior to cocaine self-administration, it is possible that neuronal-specific inhibition of CRHR1 during task acquisition resulted in alterations in initial cocaine-cue learning that may have affected the rates of extinction and performance on subsequent cue tests. Further studies of the role of CRHR1 on drug-cue learning are necessary.

CRHR1 antagonists have been suggested as a potential treatment for addiction in humans, especially alcohol use disorders (Zorrilla et al., 2013), due to the comorbidity between alcohol use disorders and anxiety disorders (Grant et al., 2004; Bruijnzeel and Gold, 2005) and polymorphisms in the Crhr1 gene related to alcohol use disorders in humans (Treutlein et al., 2006; Chen et al., 2010) and changes in Crhr1 expression related to alcohol drinking phenotypes in rodents (Hansson et al., 2006a, 2007; Molander et al., 2012). Furthermore, CRHR1 antagonists have been successful in attenuating alcohol-seeking in animal models of relapse (Hansson et al., 2006a; Marinelli et al., 2007; Sommer et al., 2008), but less successful in clinical trials (reviewed in Spierling and Zorrilla, 2017). Our findings contribute to previous data of the complex role of CRHR1 in drug-seeking behaviors, and the circumstances under which these dissociable effects occur need further evaluation in terms of CRHR1 receptor antagonists for therapeutic use. It is interesting to speculate that the effectiveness of CRHR1 antagonists to treat dependence may vary based on individual differences in CRHR1 expression.

In summary, while having no effects on cocaine-induced locomotion or primary cocaine reinforcement, we report dissociable effects of CRHR1 inactivation in DAT- and D1-containing neurons on cue-induced reinstatement following cocaine self-administration. DAT-Crhr1 mice demonstrated a significant increase in cue-induced reinstatement relative to controls, while D1-Crhr1 mice demonstrated a significant decrease in cue-induced reinstatement relative to controls, indicating neuronal selectivity of CRHR1 receptors on dopaminoceptive neurons in the control of relapse-like behavior. Thus, in addition to its well-documented role in stress-induced reinstatement of drug-seeking, CRHR1 may play an important role in cue-induced reinstatement based on neuronal localization.

## Author Contributions

REB, LB, JMD and ACH participated in the design of the studies, data collection and analysis and preparation of the article. NH participated in data collection. NJJ participated in the design of the studies. RS participated in the design of the studies, data analysis and preparation of the article.

## Conflict of Interest Statement

The authors declare that the research was conducted in the absence of any commercial or financial relationships that could be construed as a potential conflict of interest.
