# Supplementary material for: Dissociable Role of Corticotropin Releasing Hormone Receptor Subtype 1 on Dopaminergic and D1 Dopaminoceptive Neurons in Cocaine Seeking Behavior
Source: Front Behav Neurosci. 2017 Nov 13;11:221. doi: 10.3389/fnbeh.2017.00221 (PMC5693884; doi:10.3389/fnbeh.2017.00221)
Supplement: Supplementary file 1 [file Data_Sheet_1.docx]

Supplementary Material

Dissociable role of corticotropin releasing hormone receptor subtype 1 on dopaminergic and D1 dopaminoceptive neurons in cocaine seeking behavior

**Rick E. Bernardi*, Laura Broccoli, Natalie Hirth, Nicholas J. Justice, Jan M. Deussing, Anita C. Hansson, and Rainer Spanagel**

*** Correspondence:** Rick E. Bernardi: [rick.bernardi@zi-mannheim.de](mailto:rick.bernardi@zi-mannheim.de)

**Methods**

Following cue reinstatement testing, further extinction sessions were conducted until the extinction criterion was again reached, after which yohimbine-induced reinstatement was tested during a single 2 hr session in which the light CS was omitted, and was preceded 5 min prior to the beginning of the trial by an IP injection of yohimbine (2 mg/kg IP; Mantsch et al., 2010). Following yohimbine reinstatement testing, further extinction sessions were conducted until the extinction criterion was again reached, after which yohimbine + cue reinstatement was tested during a single 2 hr session that began with a 20s blinking of the light CS, and was preceded 5 min prior to the beginning of the trial by an IP injection of yohimbine (2 mg/kg IP). Yohimbine HCl (Sigma-Aldrich, Germany) was dissolved in sterile water and administered at a concentration of 10 ml/kg (Mantsch et al., 2010).

**Results**

Extinction and yohimbine reinstatement did not differ between DAT-Crhr1 mice and controls. Figure S1A and S1B show the mean (± SEM) responding on the active and inactive levers during the final 2 days of 2hr daily extinction sessions and during the 2hr yohimbine reinstatement test, respectively. A three-way ANOVA (session x lever x genotype) revealed a significant session x lever interaction [F(1,13) = 9.3, p < .05] and significant main effects of lever [F(1,13) = 14.5, p < .005] and day [F(1,13) = 5.2, p < .05], but no other effects (Fs < 1 except session x lever x genotype interaction: [F(1,13) = 1.5, p > .05]), indicating no effect of genotype across extinction and reinstatement measurements but an alteration in active and inactive lever responding across these measurements. Follow-up paired samples t-tests of the active and inactive levers revealed that active [t(14) = 3.1, p < .025, Bonferroni-corrected α=.05/2] but not inactive [t(14) = 0.4, p > .025, Bonferroni-corrected α=.05/2] responding increased significantly during yohimbine reinstatement relative to final extinction responding values, indicative of a minor yohimbine reinstatement across groups. It must be noted that DAT-Crhr1 mice demonstrated a significant increase in the number of trials required to reach the extinction criteria relative to controls [t(13) = 2.4, p < .05; for DAT-Crhr1 mice and controls, 10.3±2.9 and 3.8±0.7 extinction sessions, respectively].

Responding at the conclusion of further extinction sessions prior to yohimbine + cue reinstatement did not differ between DAT-Crhr1 mice and controls. Figure S1C shows the mean (± SEM) responding on the active and inactive levers during the final 2 days of 2hr daily extinction sessions. A two-way ANOVA (lever x genotype) revealed no main effects of lever (F < 1) or genotype [F(1,13) = 1.4, p > .05] and no significant lever x genotype interaction (F < 1). Again, DAT-Crhr1 mice demonstrated a significant increase in the number of trials required to reach the extinction criteria relative to controls [t(13) = 2.3, p < .05; for DAT-Crhr1 mice and controls, 5.9±1.7 and 2.3±0.2 extinction sessions, respectively]. During yohimbine + cue reinstatement, DAT-Crhr1 mice demonstrated higher levels of responding on the cocaine-associated active lever relative to controls, but this did not reach statistical significance. Figure S1D shows the mean (± SEM) responding on the active and inactive levers during the 2-hr yohimbine + cue reinstatement test. A two-way ANOVA (lever x genotype) revealed a significant main effect of lever [F(1,13) = 29.7, p < .001], but no main effect of genotype [F(1,13) = 2.3, p > .05] and only a trend toward a significant lever x genotype interaction [F(1,13) = 4.2, p = .06]. Despite no significant interaction, it should be noted that the values obtained during this test are very similar to those obtained during the cue reinstatement test, with DAT-Crhr1 mice demonstrating much higher yohimbine + cue reinstatement than controls.

Extinction and yohimbine reinstatement did not differ between D1-Crhr1 mice and controls. Figure S2A and S2B show the mean (± SEM) responding on the active and inactive levers during the final 2 days of 2hr daily extinction sessions and during the 2hr yohimbine reinstatement test, respectively. A three-way ANOVA (session x lever x genotype) revealed a significant session x lever interaction [F(1,17) = 8.0, p < .05] and a significant main effect of lever [F(1,17) = 16.5, p < .005], but no other effects (Fs < 1 except main effect of day: [F(1,17) = 2.5, p > .05]), indicating no effect of genotype across extinction and reinstatement measurements but an alteration in active and inactive lever responding across these measurements. Follow-up paired samples t-tests of the active and inactive levers revealed that active [t(18) = 2.4, p < .025, Bonferroni-corrected α=.05/2] but not inactive [t(18) = 0.4, p > .025, Bonferroni-corrected α=.05/2] responding increased significantly during yohimbine reinstatement relative to final extinction responding values, indicative of a minor yohimbine reinstatement across groups. It must be noted that D1-Crhr1 mice demonstrated a significant decrease in the number of trials required to reach the extinction criteria relative to controls [t(17) = 2.3, p < .05; for D1-Crhr1 mice and controls, 3.2±0.6 and 7.2±1.5 extinction sessions, respectively].

Responding at the conclusion of further extinction sessions prior to yohimbine + cue reinstatement did not differ between D1-Crhr1 mice and controls. Figure S2C shows the mean (± SEM) responding on the active and inactive levers during the final 2 days of 2hr daily extinction sessions. A two-way ANOVA (lever x genotype) revealed no significant lever x genotype interaction (F < 1) and no main effects of lever [F(1,17) = 3.7, p > .05] or genotype [F(1,17) = 1.4, p > .05]. Furthermore, D1-Crhr1 mice and controls did not differ in the number of trials required to reach the extinction criteria [t(17) = 1.0, p > .05, for D1-Crhr1 mice and controls, 2.7±0.7 and 5.5±2.5 extinction sessions, respectively]. During yohimbine + cue reinstatement, D1-Crhr1 mice demonstrated lower levels of responding on the cocaine-associated active lever relative to controls. Figure S1D shows the mean (± SEM) responding on the active and inactive levers during the 2-hr yohimbine + cue reinstatement test. A two-way ANOVA (lever x genotype) revealed a main effect of lever [F(1,17) = 50.5, p < .001] but not genotype [F(1,17) = 3.9, p > .05], but more importantly, a significant lever x genotype interaction [F(1,17) = 6.3, p < .05]. Independent samples t-tests showed that D1-Crhr1 demonstrated a trend toward a reduction in active lever pressing relative to control mice [t(17) = 2.3, p = .03, Bonferroni-corrected α=.05/2], but the two groups did not differ on inactive lever pressing [t(17) = 0.3, p > .025, Bonferroni-corrected α=.05/2]; it should be noted that the values obtained during this test are very similar to those obtained during the cue reinstatement test.

**References**

Mantsch, J. R., Weyer, A., Vranjkovic, O., Beyer, C. E., Baker, D. A., Caretta, H., 2010. Involvement of noradrenergic neurotransmission in the stress- but not cocaine-induced reinstatement of extinguished cocaine-induced conditioned place preference in mice: role for beta-2 adrenergic receptors. Neuropsychopharmacology 35, 2165-2178.

**Figure S1.**

**
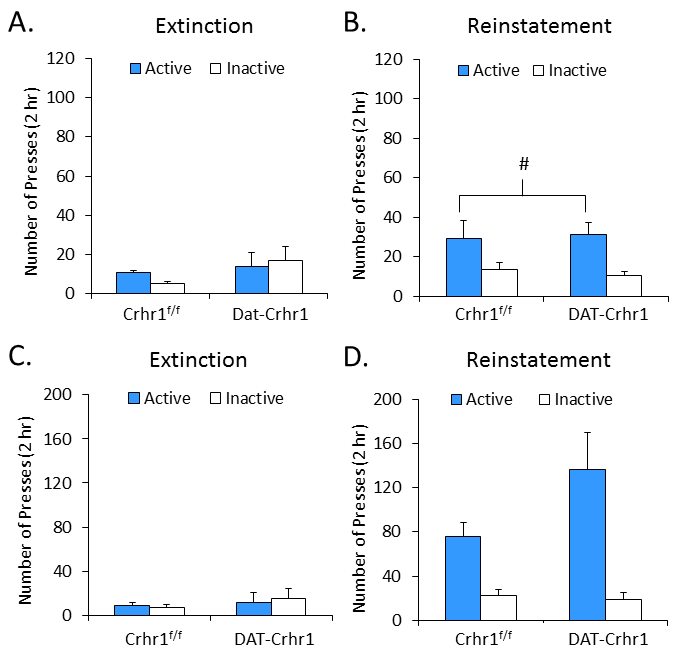
**

**Supplementary Figure 1.** Yohimbine and yohimbine + cue reinstatement in DAT-Crhr1 and littermate Crhr1^f/f^ control mice. (A) Extinction and (B) yohimbine reinstatement did not differ between DAT-Crhr1 mice. Data represent mean number of presses (± SEM) on the active and inactive levers during the final 2d of daily 2hr extinction sessions and during yohimbine reinstatement, respectively. (C) There was no difference in responding in DAT-Crhr1 mice and controls during the final two days of extinction trials prior to yohimbine + cue reinstatement. Data represent mean number of presses (± SEM) on the active and inactive levers during the final 2d of daily 2hr extinction sessions. (D) DAT-Crhr1 mice demonstrated increased cue-induced reinstatement relative to controls, but this did not reach significance. Data represent mean number of presses (± SEM) on the active and inactive levers during a single 2hr session of cue reinstatement; #p < .025 (Bonferroni-corrected α=.05/2) relative to extinction values, active lever presses

**Figure S2.**

**
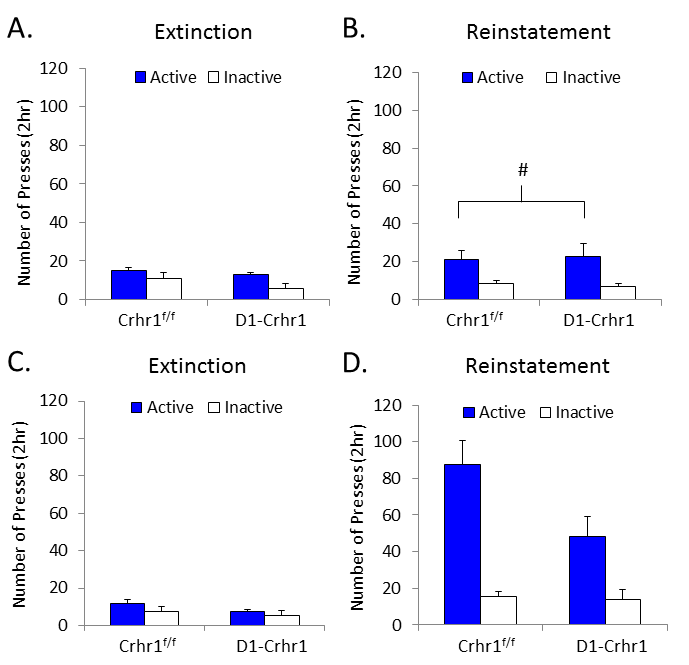
**

**Supplementary Figure 2.** Yohimbine and yohimbine + cue reinstatement in D1-Crhr1 and littermate Crhr1^f/f^ control mice. (A) Extinction and (B) yohimbine reinstatement did not differ between D1-Crhr1 mice. Data represent mean number of presses (± SEM) on the active and inactive levers during the final 2d of daily 2hr extinction sessions and during yohimbine reinstatement, respectively. (C) There was no difference in responding in D1-Crhr1 mice and controls during the final two days of extinction trials prior to yohimbine + cue reinstatement. Data represent mean number of presses (± SEM) on the active and inactive levers during the final 2d of daily 2hr extinction sessions. (D) D1-Crhr1 mice demonstrated decreased cue-induced reinstatement relative to controls, but this did not reach significance. Data represent mean number of presses (± SEM) on the active and inactive levers during a single 2hr session of cue reinstatement, #p < .025 (Bonferroni-corrected α=.05/2) relative to extinction values, active lever presses

**Figure S3.**


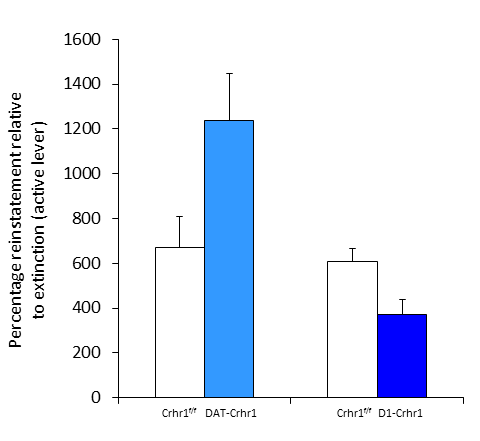


**Supplementary Figure 3.** Control mice of the DAT-Crhr1 and D1-Crhr1 lines did not differ as a function of final extinction values. There was no difference in responding on the active lever during cue reinstatement testing in control mice from the two lines relative to final extinction values. Data represent mean percentage (± SEM) of active lever responding relative to extinction during cue-induced reinstatement.
